# Genome-based Modeling and Design of Metabolic Interactions in Microbial Communities

**DOI:** 10.5936/csbj.201210008

**Published:** 2012-11-12

**Authors:** Radhakrishnan Mahadevan, Michael A. Henson

**Affiliations:** a 200 College Street, 326 Wallberg Building, Department of Chemical Engineering & Applied Chemistry, University of Toronto, Toronto, ON, M5S3E5, Canada; b Department of Chemical Engineering, University of Massachusetts, Amherst, MA 01003, United States of America

## Abstract

Biotechnology research is traditionally focused on individual microbial strains that are perceived to have the necessary metabolic functions, or the capability to have these functions introduced, to achieve a particular task. For many important applications, the development of such omnipotent microbes is an extremely challenging if not impossible task. By contrast, nature employs a radically different strategy based on synergistic combinations of different microbial species that collectively achieve the desired task. These natural communities have evolved to exploit the native metabolic capabilities of each species and are highly adaptive to changes in their environments. However, microbial communities have proven difficult to study due to a lack of suitable experimental and computational tools.

With the advent of genome sequencing, omics technologies, bioinformatics and genome-scale modeling, researchers now have unprecedented capabilities to analyze and engineer the metabolism of microbial communities. The goal of this review is to summarize recent applications of genome-scale metabolic modeling to microbial communities. A brief introduction to lumped community models is used to motivate the need for genome-level descriptions of individual species and their metabolic interactions. The review of genome-scale models begins with static modeling approaches, which are appropriate for communities where the extracellular environment can be assumed to be time invariant or slowly varying. Dynamic extensions of the static modeling approach are described, and then applications of genome-scale models for design of synthetic microbial communities are reviewed. The review concludes with a summary of metagenomic tools for analyzing community metabolism and an outlook for future research.

## 1. Introduction

Recent advances in omics and genetic engineering technologies have resulted in novel techniques to interrogate and manipulate biological processes at varying levels of detail. The availability of such tools to analyze system-wide changes at the biomolecular level of genes, proteins and metabolites has created significant opportunities to understand cellular functions and to ultimately design processes that achieve a desired objective (e.g. metabolic engineering, tissue engineering). Such tools have been increasingly used to understand, manipulate and model microbial communities for several practical applications including biorefineries, bioelectricity generation and bioremediation (32, 34).

Microbial communities are ubiquitous, and microorganisms rarely function in isolation in the environment, even though most microbiology research is focused around the study of pure culture representatives. Hence, it is valuable to investigate the interactions between microorganisms so that we can extend our understanding of the physiology of pure culture representatives to communities. Genome sequencing of whole communities in natural environments (metagenomics) is now possible (19) and a wealth of sequence data has been generated for the community members (17, 23, 43, 48, 52, 54, 55). Similarly, proteomic methods developed for single species have been extended to communities (metaproteomics) and yielded new insights into community interactions and metabolism (6, 29, 36, 51).

Extending modeling approaches to microbial communities is challenging partly due to the range of metabolic interactions that are possible, including competition, cross-feeding, syntrophy and mutualism. Additionally, the isolation of individual members of the community can be challenging despite advances in physiological techniques (3). While genome sequencing can be valuable for initial exploration of metabolism, models of community metabolism can provide additional information on the metabolic potential and the extent of metabolic interactions in microbial communities.

In this review, we focus on studies that have used genome-scale modeling approaches for analyzing and manipulating metabolism in microbial communities. We start with a brief introduction to lumped models, following which static approaches for genome-scale modeling of community metabolism are presented. Then extensions that account for community dynamics are discussed and the use of static and dynamic models for the design of synthetic communities are described. Finally, recent studies on analyzing metabolism in metagenomes are summarized and prospects for future research are discussed. This reviews aims to provide the reader with an overview of how metabolic modeling approaches are being applied for analyzing and engineering microbial communities. The reader is referred to other recent reviews (12, 15) for additional information on microbial community design, analysis and modeling.

## 2. Mathematical Models of Metabolism: From Monod to Genome-scale

The earliest attempts at developing metabolic models of individual microbes involved an unstructured and lumped approach which considered only substrate, product and biomass concentrations and used classical Monod kinetics based on a single, growth limiting substrate. Structured mathematical modeling of cellular metabolism that explicitly considered cellular compartments dates to the early 1980s when one of the first intracellular models of *Escherichia coli* was developed (8). These models were extended by the cybernetic modeling approach which accounted for metabolic regulation by explicitly considering a cellular objective (7). While cybernetic modeling has been gradually extended, and recently new hybrid approaches have been developed (46), most studies are focused on central metabolism-scale networks. The concept of a cellular objective was first proposed in the context of linear programming by Majewski and Domach (35), who used the objective of ATP maximization to explain overflow metabolism in *E. coli*. The concept of maximizing a cellular objective using linear programming was extended to the central metabolic network of *E. coli* by Varma and Palsson (50), who first developed the flux balance analysis approach where the cellular objective was assumed to be maximization of growth rate. Tradeoffs between the objectives of metabolic efficiency and rates have been explored in other papers (38, 39, 42, 45) and are not discussed extensively in this review.

The advent of genome-sequencing technologies and bioinformatics allowed the reconstruction of large-scale metabolic networks in model organisms, which paved the way for the extension of flux balance analysis to genome-scale metabolic networks (10). In addition, a range of methods to interrogate these large-scale metabolic networks using linear optimization techniques collectively known as constraint-based modeling were developed (2). These developments allowed the construction of the first genome-scale models of *Escherichia coli* (10) and *Saccharomyces cerevisiae* (14) in the early 2000s. Subsequently, experimental results from chemostats validated many predictions of the *E. coli* genome-scale model, including the optimal growth rate (9) and the end-point of adaptive evolution (13, 27). These results suggested that the use of a suitably chosen cellular objective might be sufficient to overcome the lack of metabolic regulation represented in such genome-scale models. However, recently it has been shown that a combination of growth maximization and minimization of adjustment from a reference metabolic state might be a more appropriate objective function for capturing the intracellular flux distribution (42). While these genome-scale metabolic models have been extensively used to facilitate metabolic engineering through the development of novel bilevel optimization algorithms, we do not discuss these developments in this review and instead refer the interested reader to another review (4).

The aforementioned studies are based on static metabolic models that assume the intracellular and extracellular states are time invariant. To overcome this limitation, dynamic extensions of the constraint-based modeling approach, collectively termed dynamic Flux Balance Analysis (dFBA), have been developed. The dFBA approach links the steady-state intracellular metabolic flux distribution with the dynamic changes in the environment and allows the prediction of microbial growth, substrate utilization and product formation dynamics. Initially, dFBA was used to model the diauxic growth of *E. coli* on mixtures of multiple substrates (5, 33, 50). More recently, dFBA has been extended to fed-batch optimization (24) and *in silico* metabolic engineering (25) of *S. cerevisiae* cultures. Next, we will focus on how genome-scale models of metabolism have been used to investigate metabolic interactions in synthetic and natural communities of microbes.

## 3. Static Genome-scale Models of Microbial Communities

### 3.1 Overview

Microbial communities often involve species interactions where a metabolic by-product of one species is the substrate for other species and vice versa. In the environment, carbohydrates from organic matter are fermented to hydrogen, acids and alcohols by fermentative microbes and these electron donating byproducts are oxidized with electron acceptors such as Fe(III), sulfate and CO_2_ by metal reducers, sulfate reducers and methanogens, respectively. Hence, models of community metabolism have to account for the environment as a separate compartment in addition to the intracellular metabolic networks of individual organisms in the community. Most often, these interacting partners are assumed to grow together and this metabolic interaction is assumed to be at steady state. There have been several such “static genome-scale models” of microbial communities that are summarized in the next section.

### 3.2 Representative studies

One of the first multi-species metabolic models considered mutualistic interactions between the sulfate reducer, *Desulfovibrio vulgaris*, and the methanogen, *Methanococcus maripaludis* (47). *D. vulgaris* typically uses lactate as a substrate and secretes a mixture of formate, hydrogen, acetate and CO_2_ in the absence of sulfate, while *M. maripaludis* consumes acetate, hydrogen and CO_2_ to produce methane. The study utilized small-scale metabolic models of core primary metabolism, with *D. vulgaris* represented by 86 reactions and 75 metabolites and *M. maripaludis* represented by 82 reactions and 72 metabolites. The authors assumed that all the formate and hydrogen secreted by *D. vulgaris* was completely consumed by *M. maripaludis* and used a community objective that weighted the *D. vulgaris* and *M. maripaludis* growth rates in a 10:1 ratio. The predicted growth and metabolic flux distribution were found to be consistent with experimental data, and the simulations suggested that hydrogen exchange was essential for mutualistic growth but formate was not essential.

An example of the use of genome-scale models for analyzing metabolic interactions in communities is described in Wintermute and Silver (2010) (53). The authors considered 46 conditionally lethal auxotrophic strains of *E. coli* and evaluated with co-culture experiments the ability of these mutants to sustain growth in 1035 pairs. They found that in 17% of cases metabolic synergy was obtained, where one strain synthesized a metabolite that was essential for the second strain and vice-versa to allow both strains to grow in the absence of their respective essential metabolite in the media. They then used a genome-scale model of *E. coli* to calculate a co-operation metric based on the ratio of the predicted cost of synthesizing the donated metabolite for first strain and the growth benefit in the second strain from the metabolite received. The calculated co-operation metrics were found to be consistent with experimentally measured improvements in the strain growth rates, suggesting that genome-scale metabolic models can be valuable in quantifying the fitness costs and benefits of metabolic interactions in microbial communities.

Freilich and Ruppin (2011) (16) extended the analysis of metabolic interactions to 6,903 species pairs derived from 118 bacteria for which genome-scale metabolic models were automatically constructed using the Model SEED platform (22). The authors provided a conceptual framework for predicting three possible interactions depending on how the sum of the growth rates when grown together (co-growth) compared with the sum of the growth rates when grown separately in the same media. This framework included: 1) a no interaction case, where the co-growth rate was the same as the sum of the individual growth rates; 2) a competition case, where the co-growth rate was lower; and 3) a co-operation case, where the co-growth rate was higher. Since these interactions were highly condition dependent, methods to modify the media so as to induce competition or co-operation were developed. The authors then tested the computational predictions of interactions experimentally using 10 bacterial pairs across three media conditions (original, competition inducing and co-operation inducing). The interactions were found to be correctly predicted in 65% of the 30 experiments. Finally, the authors used ecological data from 2,801 samples collected from 59 niches and predicted competition and co-operation in these bacterial species pairs. They found that mean competition and co-operation metrics were higher in these samples than in ecologically non-associated pairs. Based on these results, the authors suggested that the analysis of genome-scale models could be valuable in identifying ecological principles that govern metabolic interactions in complex natural environments.

In most studies, the community objective has been assumed to be growth rate maximization of the individual microbes. Recently, Zomorrodi et al. (2012) (58) formulated a bi-level optimization problem (OptCom) that considered a community level objective function in addition to the individual species objective function of growth rate maximization. Using the OptCom framework, the authors were able to modify the community level objective function to represent a variety of metabolic interactions including syntrophy, cross-talk (bi-directional metabolite exchange), synergism, commensalism, parasitism and competition. The framework was then used to analyze metabolic interactions in a phototrophic bacterial mat and in a synthetic community consisting of *Desulfovibrio vulgaris, Geobacter sulfurreducens* and *Clostridium cellulolyticum*. The predictions of carbon and electron balance were found to be consistent with experimental observations. The authors also investigated a hypothetical scenario where new species were added to this microbial community and methane production was simulated. These results suggest the potential for a model-based framework in designing synthetic microbial communities for practical applications in bioenergy and bioremediation.

## 4. Dynamic Genome-scale Models of Microbial Communities

### 4.1 Overview

While the static models described in the previous section have been shown to be valuable for understanding and analyzing metabolic interactions in microbial communities, the static approach does not allow prediction of the biomass concentrations of the individual species or account for time-varying changes in the intracellular and extracellular environments. Consequently, static genome-based models cannot easily predict the microbial composition in dynamic environments. Recently, dynamic flux balance analysis (dFBA) has been extended to microbial communities to predict time-varying interactions between species and their effects on the microbial composition. The extension involves coupling genome-scale metabolic models of the individual species, dynamic extracellular mass balances on each species, substrate and metabolic byproduct, and uptake kinetics for each substrate/species pair. Below several representative studies are summarized to illustrate the potential of the dFBA approach.

### 4.2 Representative studies

Typically, a diversity of microorganisms compete for resources and/or interact cooperatively and these interactions influence the microbial composition in the environment. Transient prediction of the individual species concentrations requires dynamic models. Zhuang et al. (2010) (56) have developed a Dynamic Multi-Species Metabolic Modeling framework that integrates genome-scale metabolic models within the dFBA framework to predict metabolic flux distributions and biomass concentrations of the individual species as well as the substrate and product concentrations in the extracellular environment. This method allows multiple constraint-based metabolic models to share and exchange metabolites with each other and with the environment and is capable of dynamically predicting community growth and metabolism. The authors used this framework to evaluate the outcome of competition between two metal reducers, *Rhodoferax* and *Geobacter*, in natural and acetate amended environments. In agreement with data, the simulations suggested that *Geobacter* species were fast growing, energetically inefficient organisms relative to the *Rhodoferax* species and would dominate the community at high acetate availabilities. The *Rhodoferax* species were predicted to be most efficient at utilizing acetate and to be dominant during acetate limitation. Salimi et al. (41) used this framework to analyze metabolic interactions between *Clostridium cellulolyticum* and *Clostridium acetobutylicum*. They developed a dynamic genome-scale model of the co-culture and used the framework to understand the mechanisms behind the enhanced cellulose degradation in the co-culture relative to a monoculture of *C. cellulolyticum*. They varied the strength of cellobiose inhibition on cellulose solubilization, and the simulation results suggested that the cellobiose inhibition was not the primary factor underlying the synergistic enhancement of cellulose solubilization in the co-culture.

Sequential uptake of hexose and pentose sugars derived from cellulosic biomass limits the ability of pure microbial cultures to efficiently produce renewable bioproducts. Hanly and Henson (20) have utilized the dFBA framework to investigate the capability of mixed cultures of substrate-selective microbes to improve the utilization of glucose/xylose mixtures and to convert these mixed substrates into products such as ethanol. In the first study, batch co-culture simulations were performed with *E. coli* mutant strains ALS1008 and ZSC113, which have been engineered to have glucose and xylose only uptake, respectively. The simulations suggested that improvements in batch substrate consumption observed in a previous experimental study (11) resulted primarily from an increase in ZSC113 xylose uptake relative to wild-type *E. coli*.

In the same study, the xylose only consuming strain ZSC113 was computationally co-cultured with wild-type *S. cerevisiae*, which can only uptake glucose. Under the simplifying assumption that both microbes grew optimally at the same pH and temperature, simultaneous optimization of the initial ZSC113/*S. cerevisiae* ratio and the oxygenation level through the batch produced an almost two-fold increase in predicted ethanol productivity compared to pure cultures of wild-type *E. coli*. In a combined experimental/computational study of the ZSC113/*S. cerevisiae* co-culture system (21), the same authors demonstrated that the two genome-scale models could be adapted to suboptimal, common temperature and pH simply by adjusting the non-growth associated ATP maintenance of each model. The inhibitory effect of ethanol produced by *S. cerevisiae* on ZSC113 xylose uptake was found to be the only interaction necessary to include in the co-culture model to match measured species, substrate and ethanol concentration profiles. Furthermore, the co-culture model successfully predicted initial ZSC113/*S. cerevisiae* ratios that resulted in simultaneous glucose and xylose exhaustion for different sugar mixtures.

## 5. Model-based Engineering of Synthetic Microbial Communities

**Figure 1:**
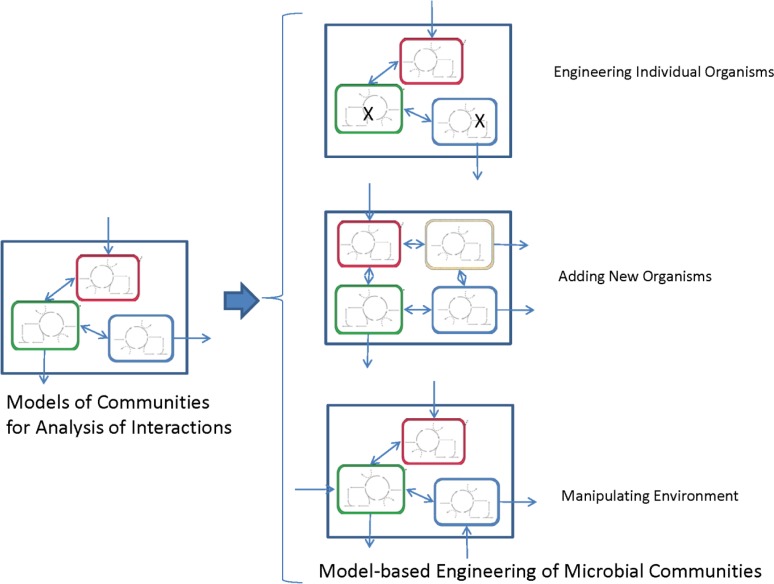
Genome-scale models for analyzing and engineering metabolic interactions in microbial communities.

While the studies described in the previous sections are valuable for analyzing species interactions, there have been comparatively few reports where genome-scale models have been used to identify potential strategies for engineering metabolism in communities. Typically, engineering communities can involve: 1) engineering the metabolism of individual microbes in defined microbial communities; 2) manipulating the community by adding new microbes (e.g., bioaugmentation); and 3) engineering the environment by introducing substrates that allow a specific species of interest to dominate (e.g., bioremediation) (Figure 1). Below studies that have used genome-scale models to identify strategies for engineering communities are detailed.

### 5.1 Engineering Community Members and Composition

Tzamali *et al*. (49) analyzed the metabolism of all viable single gene deletion mutants of *E. coli* across 58 different carbon sources using a dFBA approach. They computed the maximum concentrations of the secreted metabolites for each scenario and used this information to define an interaction metric for a pair of mutants growing in a single carbon source environment. The metric was defined as the largest difference between the maximal byproduct concentrations scaled by the maximum concentration of byproduct amongst the two strains. The authors then used graph theoretic approaches to identify metabolic interaction networks, and propose several mutant pairs that could exchange metabolites, and identify examples of both commensalistic and cross-talk interactions. However, none of the predicted interactions have yet been verified experimentally. Nevertheless, such experimental evaluation of synthetic co-operation in genetically modified yeast strains has been performed (44) and these results indicate the possibility of explicitly engineering co-operative interactions in *E. coli, S. cerevisiae* and other organisms for which well curated, genome-scale metabolic models and genetic engineering tools are available.

In addition to *in silico* engineering of metabolism in individual organisms, genome-scale community models can be used to investigate the systematic introduction of new species within a particular environment. For example, Zomorrodi et al. (58) used their OptCom modeling framework to evaluate the effect of introducing a methanogen into an existing community and found that methane production could be increased if additional hydrogen was present. Taken together, these studies demonstrate the value of using genome-scale metabolic models to engineer metabolic interactions in communities. Finally, genome-scale models can be used to manipulate the community composition to optimally perform a given task. Applying dFBA to a synthetic consortium consisting of the *E. coli* mutant ZSC113 and wild-type *S. cerevisiae*, Henson and Hanly (21) showed that a dynamic community model successfully predicted inoculum compositions that produced simultaneous glucose and xylose exhaustion for different sugar mixtures in batch culture.

### 5.2 Engineering Microbial Community Environment

Several experimental studies have highlighted the value of manipulating the environment through spatial segregation for creation of co-operative metabolic interactions (39). For example, Kim et al. (28) showed that spatial segregation of a pentachlorophenol (PCP) degrader (*Sphingobium chlorophenolicum*) and a mercuric ion reducer (*Ralstonia metallidurans*) was required to degrade PCP in the presence of mercury as *S. chlorophenolicum* was sensitive to mercuric ion. Following a different approach, Klitgord and Segre (30) have used genome-scale metabolic models to identify environmental conditions (carbon and nitrogen sources) that result in different metabolic interactions (neutral or no interaction, commensalism or one way interaction, mutualistic or two way interaction) and test this approach on pairs of microorganisms including mutant *S. cerevisiae* and *E. coli* strains. The predicted interactions can occur in 4.7% of 11.6 million cases tested. In addition, the authors have applied the same methodology to genome-scale models of seven different microorganisms and identified media conditions that enabled a range of metabolic interactions.

Currently there are few reported examples of utilizing dynamic genome-scale metabolic models for designing the community environment. Zhuang et al. (57) have used a dynamic genome-based model of a microbial community consisting of *Geobacter sulfurreducens* and *Desulfobacter* to identify the optimal acetate and Fe(III) injection rates to allow sustained uranium bioremediation to occur in the environment. For the ZSC113/*S. cerevisiae* co-culture system, Hanly and Henson (21) used genome-scale metabolic models to determine the optimal inoculum composition and aerobic-to-anaerobic switching time for batch ethanol production from glucose/xylose mixtures.

## 6. Summary and Outlook

The studies reviewed in this paper clearly demonstrate the value of genome-scale models for understanding and engineering metabolic interactions in microbial communities. The availability of such rigorous and experimentally validated models will provide the opportunity to systematically optimize microbial communities for practical applications through the engineering of the individual species, addition of new species to existing communities and manipulation of the community composition and environment (Figure 1). Individual species engineering will require the availability of genetic tools for the organism of interest, and this requirement will generally limit metabolic engineering to model species. Furthermore, detailed physiological data on the response of the community to different perturbations will be required to validate the modeled interactions. A potential validation approach would be the systematic introduction of perturbations such as the addition of a new substrate or the introduction/elimination of a community member and assessment using systems biology tools. Obtaining such data might also necessitate the isolation of individual organisms in environmental samples, which may impede genome-scale model construction. The characterization of dominant community members can lead to a representative model that adequately captures the collective metabolism of the more complex community. Another limitation of this genome-scale modeling approach is that the models are deterministic and cannot capture stochastic processes that may affect the outcome of microbial competition.

While this review has focused on organisms for which an annotated genome sequence is available, metabolic network reconstructions of metagenomes are increasingly possible. Such reconstructions of overall communities have been applied to environmental metagenomes and human microbiomes. For example, a metabolic network reconstructed from the metagenome of the English Channel has been used to define a metric that correlated the presence of metabolic enzymes with the metabolic turnover of the associated pathways (31). Additionally, the metabolic networks of three dechlorinating metagenomes have been compared and used to identify functional redundancies in key metabolic pathways that synthesize co-factors and amino acids which contribute to the robustness of community function (26).

In the future, we anticipate that such genome-scale models can be generalized to more than three species and therefore can be extended to the main members of natural communities. In addition, integration of these microbial community models with biogeochemical, physical processes in ecosystem models will allow improved prediction of community responses to environmental perturbations. Game theoretic approaches have been proposed to represent both the fitness of the individual members and changes in the environment due to microbial dynamics (38, 40). The integration of such game theoretic descriptions with genome-scale models represents a potential opportunity to improve the accuracy of microbial community modeling methods. Model-based analyses have also been applied to the human gut microbiome. For example, metabolic networks from the gut microbiome of 124 individuals were used to identify differences in metabolism associated with obesity and inflammatory bowel disease (18). It is expected that advances in bioinformatics and metabolic network modeling will enable the application of such methods for in-depth analyses of the entire gut microbiome (1, 37) and for connecting whole body metabolism with the metabolic potential of the microbiome.
